# Patient satisfaction with the management of refractory and unexplained chronic cough in Canada: Results from a national survey

**DOI:** 10.1371/journal.pone.0308275

**Published:** 2024-08-01

**Authors:** Sana Khan, Danica Brister, Ted Abraham, Samuel Laventure, Sevag Sahakian, Berta Juliá, Imran Satia

**Affiliations:** 1 Department of Medicine, McMaster University, Hamilton, Ontario, Canada; 2 Medical Affairs, Merck Canada Inc., Kirkland, Quebec, Canada; 3 Medical Department, MSD, Madrid, Spain; 4 Firestone Institute for Respiratory Health, St. Joseph’s Healthcare, Hamilton, Ontario, Canada; University of Cape Coast College of Health and Allied Sciences, GHANA

## Abstract

**Background:**

Chronic cough (persisting for ≥8 weeks) is a common disorder affecting approximately 5 to 10% of adults worldwide that is sometimes refractory to treatment (refractory chronic cough [RCC]) or has no identifiable cause (unexplained chronic cough [UCC]). There is minimal information on the patient’s experience of RCC/UCC in Canada. The aim of this study was to evaluate the patient journey and perceptions related to RCC/UCC management in Canada.

**Methods:**

Our exploratory study included Canadians in the Leger Opinion Panel and focused on individuals with RCC or UCC. Key entry criteria were: age ≥18 years, cough on most days for ≥8 weeks, no smoking within 1 year, no serious respiratory disease or lung cancer, and not taking angiotensin-converting enzyme inhibitors. Individuals who met entry criteria were invited to complete an approximately 30-minute online survey with questions on demographic characteristics, healthcare professional (HCP) interactions, diagnosis of underlying conditions, current treatments, and satisfaction with HCPs and chronic cough therapies.

**Results:**

A total of 49,076 individuals completed the chronic cough screening questionnaire (July 30, 2021 to September 1, 2021): 1,620 (3.3%) met entry criteria for RCC or UCC, and 1,046 (2.1%) completed the online survey (mean age of 45 years, 61% female). Most respondents (58%) reported their chronic cough was managed by a general practitioner (GP). Forty-four percent of respondents did not have a diagnosis of an underlying condition for their cough. Breathing tests (39%) and chest imaging (34%) were the most common diagnostic tests. Cough suppressants (18%) were the most frequent current treatment. Respondents were moderately satisfied with their HCPs, but more than half considered their treatment ineffective and 34% had considered no longer seeking medical attention because of a lack of treatment success.

**Conclusions:**

Individuals with RCC/UCC in Canada are largely unsatisfied with the effectiveness of treatment. Additional HCP education and new treatment options are needed to improve patient satisfaction.

## Introduction

Approximately 16% to 18% of Canadians aged 45 to 85 years [1] and perhaps 10% of the population worldwide [2] have chronic cough, defined as a cough that persists for more than 8 weeks [3, 4]. Chronic cough negatively impacts health-related quality of life (HR-QoL) and results in reduced work productivity [5–9]. Despite the prevalence of this condition, recent surveys of healthcare professionals in Canada [10, 11] and elsewhere [12–14] have revealed limited knowledge of current guidelines and management strategies for chronic cough.

The European Respiratory Society (ERS) [3] and American College of Chest Physicians [4, 15] have published guidelines for the management of chronic cough. There are currently no Canadian-specific guidelines developed with a GRADE/PICO evidence-based approach for the management of chronic cough, but there is a recent Canadian consensus review article [16] that summarizes chronic cough guidance and is consistent with guidelines published by the ERS [3]. In general these guidelines have four major principles: (1) exclude and treat obvious causes; (2) investigate and treat common triggers of chronic cough; (3) exclude and treat rarer triggers of chronic cough; (4) manage unexplained chronic cough with speech therapy, low-dose morphine, and/or neuromodulator treatments (amitriptyline, gabapentin, pregabalin) [17].

Diagnostic tests play a key role in uncovering underlying etiologies of chronic cough, such as chronic obstructive pulmonary disease (COPD), gastroesophageal reflux disease (GERD), or use of angiotensin-converting enzyme (ACE) inhibitors [18–20], but recent studies have found that even after thorough investigation and treatment, 20% to 40% of chronic cases do not respond to guideline-based treatment [21, 22]. These cases, referred to as “refractory chronic cough” (RCC; chronic cough that persists despite optimal treatment of an underlying condition) or “unexplained chronic cough” (UCC), can be particularly difficult to manage [3, 4, 16]. Recent studies suggest that patients with RCC and UCC share demographic and clinical characteristics, particularly with respect to cough hypersensitivity, which has been proposed to play an underlying role in RCC/UCC manifestations [23–26]. Cough hypersensitivity is characterized by increased neural responsitivity to normally innocuous stimuli, which may be mediated through central neural networks [27, 28]. In addition to sharing a tendency toward cough hypersensitivity, there is evidence that the RCC/UCC populations may also respond similarly to new therapies for chronic cough [23], and some experts have suggested that from a management perspective these diagnoses may represent a single patient population [25].

A common finding in studies of chronic cough throughout the world is the frustration patients experience with their journey and their perception of lack of effectiveness of available management strategies [7, 9, 18, 29–32]. In one study, about half of patients were receiving treatment that was deemed “not effective.” [21]. However, little is known about the satisfaction of individuals with the management of RCC/UCC in Canada. As different countries vary considerably in medical care frameworks and access, extrapolation of the patient experience from other countries may be misleading. Thus, the aim of this exploratory study was to evaluate self-reported healthcare professional (HCP) interactions, diagnosis of underlying conditions, treatments, and satisfaction with the management of RCC/UCC in Canadian individuals. In a companion manuscript [33], we use responses from this survey to evaluate the patient burden associated with current ongoing RCC/UCC. The aim of both analyses was to provide a better understanding of the burden of chronic cough, particularly RCC and UCC, on Canadian individuals and society.

## Methods

### Study participants

Our exploratory study included Canadians voluntarily enrolled in the Leger Opinion Panel, a market research and analytics database that contains data from individuals throughout Canada. Most (70%) panel members are recruited by a call center, and approximately 30% are recruited via invitation and affiliate programs, partner campaigns, offline recruitment, and other methods. All panel members go through screening procedures to validate the participant’s identity and prevent duplicate accounts and fraud. Members of this panel were offered the opportunity to participate in the survey reported here; participation was voluntary and anonymous. Informed consent was obtained electronically from all respondents by an opt-in checkbox prior to study participation (see S1 Methods for the exact consent statement). Minors were excluded from the study. All data were self-reported on an electronic online survey. Respondents remained fully anonymous. The survey period began on July 30, 2021 and ended on September 1, 2021. There was no separate recruitment period.

To better characterize satisfaction of patients with management of RCC/UCC, participants were first given a screening questionnaire and required to meet the following entry criteria:

- Age of 18 years or older
- Experienced a cough on most days for the **past 8 weeks or longer**
- Not currently smoking cigarettes, marijuana, or other tobacco products, including e-cigarettes or other vaping devices, or must have quit at least 1 year ago
- Former smokers must not have smoked ≥20 pack years while smoking (a pack year is equivalent to the average of one pack per day for one year)
- No prior diagnosis of idiopathic pulmonary fibrosis (IPF), COPD, or lung cancer
- Not currently taking ACE inhibitors to treat high blood pressure, heart problems or other conditions
- Not diagnosed with COVID-19 within the past 6 months or started experiencing cough after being diagnosed with COVID-19
- Not currently participating in any clinical trial(s) sponsored by a pharmaceutical company

### Study design and online survey

Individuals who met the inclusion criteria were sent a link to an approximately 30-minute online survey with questions on demographic characteristics, including comorbidities, and questions concerning HCP interactions related to chronic cough, diagnostic tests, treatments and satisfaction with their chronic cough management (see S1 Methods for the full questionnaire). The survey was available in English or French. Questions were developed in consultation with experts in the management of chronic cough and consisted of multiple-choice questions or questions with answers rated on a 7-point Likert scale, ranging from 1 (worst) to 7 (best) (see Results section and figures for specific questions and reporting scales).

The study protocol and questionnaire were reviewed and approved by the Veritas Independent Review Board prior to study initiation (May 18, 2021; tracking number 2021-2691-6128-3).

### Statistical analysis

Because this was an exploratory study, no formal sample size calculations were performed. The target sample size for this study was 1,000 individuals with RCC/UCC as per the entry criteria to allow a robust population for data analysis. The study enrolled a non-probability sample and descriptive statistics were performed on anonymized data. All analyses of descriptive data were conducted on observed data; missing data were not imputed.

## Results

Between July 30, 2021 and September 1, 2021, 49,076 Leger Opinion Panel participants completed the chronic cough screening questionnaire and 6,563 respondents (13.3%) reported currently experiencing a cough most days for the past 8 weeks. One thousand six hundred twenty Canadians (3.3% of all participants and 24.7% of participants with self-reported chronic cough) met entry criteria for RCC/UCC (proportions of patients excluded for each entry criterion are reported in our companion manuscript [33]). A total of 1,046 individuals (2.1% of total participants, 15.9% of respondents with chronic cough, and 64.6% of eligible participants with chronic cough) completed the online survey.

### Characteristics of survey respondents

The mean age of the 1,046 eligible respondents was 45 years, 61% were female, and 23% were former smokers (stopped more than 1 year ago) (Table 1). Respondents reported having had a chronic cough daily or almost daily for a mean (standard deviation [SD]) of 5.3 (9.1) years and 28% considered their health to be fair or poor. The most common comorbidities possibly contributing to chronic cough were asthma (25%) and gastroesophageal reflux disease (GERD) (15%). Current diagnoses of chronic pain (12%) and obesity (11%) were also common.

**Table 1:** Characteristics of Canadian individuals who completed an online chronic cough survey.

Characteristic	Value
Number of individuals completing the survey	1,046
Mean age (SD), years	45 (17.1)
Female, n (%)	636 (61%)
Ethnicity, n (%)^a^	
White/Caucasian	775 (74.1%)
Chinese	86 (8.2%)
South Asian (e.g. East Indian, Pakistani, Sri Lankan, etc.)	56 (5.4%)
Southeast Asian (e.g. Vietnamese, Cambodian, Malaysian, etc.)	40 (3.8%)
Black	22 (2.1%)
Indigenous	19 (1.8%)
Latin American	14 (1.3%)
Other	36 (3.4%)
Prefer not to answer	25 (2.4%)
Mean cough duration (SD), years	5.3 (9.1)
Former smoker (including cigarettes, other tobacco products, marijuana, and e-cigarettes/vaping), n (%)	239 (23%)
Current diagnosis of condition possibly associated with chronic cough, n (%)	
Any (asthma, GERD, or UACS; respondents could have more than one)	402 (38%)
Asthma	263 (25%)
GERD	156 (15%)
UACS	17 (2%)
None of the above	644 (62%)
Current diagnosis of other comorbidities (≥5% of respondents)	
Chronic pain	122 (12%)
Obesity	113 (11%)
Rheumatologic disease	96 (9%)
Thyroid disease	82 (8%)
Diabetes without chronic complications	70 (7%)
Bladder problems	51 (5%)
Current health status, n (%)	
Poor	43 (4%)
Fair	248 (24%)
Good	457 (44%)
Very good	245 (23%)
Excellent	53 (5%)
Community size, n (%)	
<10,000 inhabitants	148 (14%)
10,000 to <100,000 inhabitants	153 (15%)
100,000 to 500,000 inhabitants	252 (24%)
>500,000 to <1,000,000 inhabitants	166 (16%)
>1,000,000 inhabitants	327 (31%)
Household income, n (%)	
<$40,000	194 (19%)
$40,000 to <$80,000	292 (28%)
$80,000 to <$100,000	165 (16%)
>$100,000	299 (29%)
Prefer not to answer	96 (9%)
Drug insurance coverage	
Public/provincial	472 (45%)
Private	459 (44%)
No coverage	64 (6%)
Don’t know	36 (3%)
Prefer not to answer	15 (1%)

^a^Some respondents chose more than one answer.

Percentages may not total 100% due to rounding.

GERD, gastroesophageal reflux disease. UACS, upper airway cough syndrome

### Consultations on RCC/UCC with healthcare professionals and other sources

A total of 633 respondents (61%) reported the time between the start of their cough and their first interaction with a doctor or nurse related to the cough; the remaining 413 (39%) did not know. Of respondents who listed a time interval, the median time between the start of their cough and their first healthcare interaction related to the cough was 1.4 months. Factors that prompted the first HCP interaction included patient decision (47%), encouraged by family/friends (15%), noticed by doctor during consultation for another issue (14%), and encouraged by caregiver (2%).

**Fig 1:**
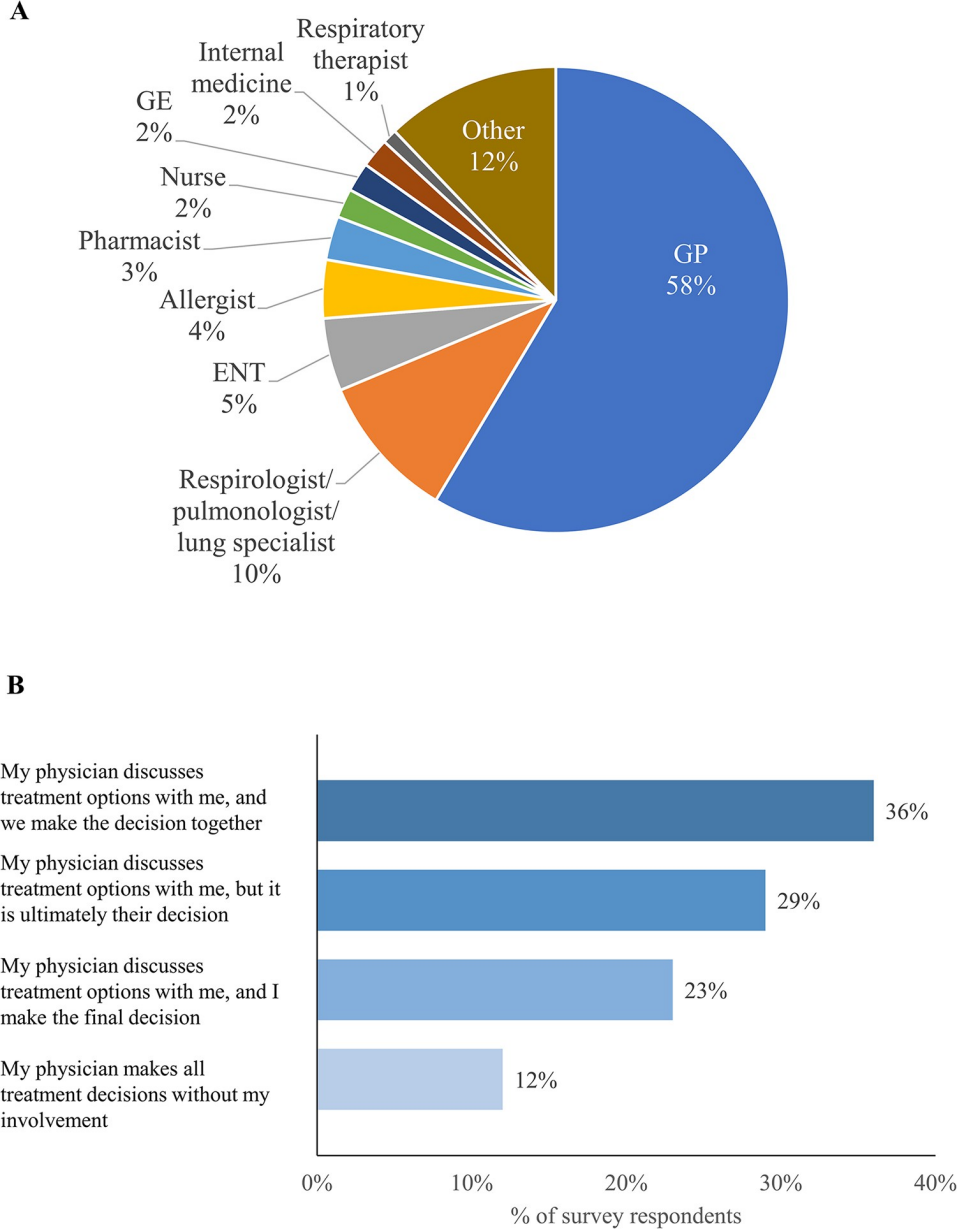
(A) Current main healthcare professionals for chronic cough and (B) involvement of respondents in treatment decisions. For (A), respondents were asked “Who do you consider to be the main person responsible for the treatment and management of your cough at this time?” For (B), respondents were asked “Overall, how would you describe your involvement in treatment decisions for your chronic cough?” ENT, ear, nose, and throat specialist. GE, gastroenterologist.

GPs were the HCP most frequently consulted for RCC/UCC (68%) (Table 2) and were most commonly considered the main person responsible for the treatment and management of the respondent’s cough at the time of the survey (58%) (Fig 1A), followed by respirologists/pulmonologists/lung specialists (22% ever consulted, 10% currently managing condition). Only 34% of patients reported having been informed or diagnosed with an underlying condition related to their cough; this required an average of 2 HCPs and 4 visits to receive such information.

**Table 2:** HCPs and other sources ever consulted by Canadian respondents with RCC/UCC.

Healthcare professional or source	% of respondents who consulted n (%) N = 1,046	Mean number of visits in last 3 months^a^	Mean rating for usefulness of information rating^a^^,^^b^
Healthcare professional			
General practitioner	709 (68%)	0.9	4.4
Respirologist/pulmonologist/lung specialist	226 (22%)	1.0	5.2
Allergist	195 (19%)	0.6	4.6
ENT	170 (16%)	1.0	4.2
Pharmacist	87 (8%)	1.4	4.9
Respiratory therapist	67 (6%)	0.7	5.0
Nurse	63 (6%)	1.3	4.5
Naturopath^c^	59 (6%)	NA	5.0
Gastroenterologist	57 (5%)	0.6	4.4
Internal medicine doctor	40 (4%)	1.0	4.5
Homeopath^c^	26 (2%)	NA	4.9
Physiotherapist	21 (2%)	2.5	4.3
Urologist	19 (2%)	2.6	4.4
Speech and language therapist	19 (2%)	1.4	5.0
Head and neck surgeon	7 (1%)	2.3	5.4
Other	134 (13%)	0.6	4.0
Other sources			
General online internet searches	384 (37%)	NA	4.8
Family or friends	229 (22%)	NA	4.7
Other chronic cough sufferers	54 (5%)	NA	4.9
Patient associations (including their website)	46 (4%)	NA	5.0
Other	30 (3%)	NA	4.5

^a^For patients who consulted the specified HCP or source

^b^On a 7-point scale where 1 = not useful and 7 = extremely useful

^c^Categorization as a healthcare professional may differ depending on local regulations

ENT, ear, nose, and throat specialist. HCP, healthcare professional. NA, not applicable. RCC, refractory chronic cough. UCC, unexplained chronic cough.

Of HCPs consulted by at least 50 respondents, the ones with the most favorable ratings as a useful source of information for RCC/UCC were respirologists/pulmonologists/lung specialists (mean rating of 5.2 on a 7-point scale from 1 = not useful to 7 = extremely useful) followed by respiratory therapists and naturopaths (both 5.0) (Table 2). Head and neck surgeons (5.4) and speech and language therapists (5.0) also had high ratings, but were only ever consulted by 7 and 19 patients, respectively. The mean rating for the usefulness of information from GPs was 4.4. Overall, differences in usefulness ratings were modest. Most respondents (88%) agreed that their physician discussed treatment options with them (Fig 1B).

With respect to non-HCP sources consulted, 37% of respondents had conducted general internet searches and 22% had consulted their family or friends. Other sources were uncommon (fewer than 60 respondents [6%]). Consultations from non-HCP sources were given generally high ratings for usefulness (mean scores of 4.5 to 5.0) (Table 2).

At the time of the survey, 20% of respondents were currently waiting for an appointment with a specialist related to their cough, and 34% stated they had at some point chosen to stop seeking further medical attention for their cough due to lack of success with treatment to date.

### Diagnosis of underlying conditions for RCC/UCC

To provide additional information on diagnoses relevant to RCC/UCC, respondents were asked to report how long it took after the initial visit to receive a diagnosis of the underlying cause for the cough OR confirmation that it was not due to a serious condition. Of the 56% of respondents who had received a diagnosis for an underlying condition related to their cough or confirmation that it was not due to a serious underlying condition, the diagnosis or “rule out” was received within 3 months of the initial HCP visit for 36% of patients, within 3 months to a year for 14%, and greater than a year for 6%. Delays were mainly due to monitoring of symptoms (41%), waiting for a specialist referral (18%), or waiting for additional tests (15%). Forty-four percent of respondents did not have an underlying diagnosis for their chronic cough or did not have confirmation that it was not due to a serious condition.

Breathing tests (lung function/spirometry) (39%), chest imaging (34%), blood tests (31%), and allergy tests (26%) were the most commonly performed diagnostic tests for evaluation of RCC/UCC. Other diagnostic tests were performed in fewer than 15% of respondents (Table 3). However, a substantial proportion of respondents (33%) reported that they did not know if they had undergone any diagnostic tests or had not undergone any of the listed options. For patients in whom diagnostic tests were performed, repeat testing was common, particularly for blood tests (48%), breathing tests (44%), and chest imaging (38%) (Table 3).

**Table 3:** Diagnostic tests performed for RCC/UCC.

Characteristic	Tests ever completed n (%) N = 1,046	Repeated tests among respondents who ever completed the test n (%)
Breathing tests (lung function/spirometry)	404 (39%)	178 (44%)
Chest imaging (CT scan/X-ray)	356 (34%)	135 (38%)
Blood test	326 (31%)	158 (48%)
Allergy test	272 (26%)	92 (34%)
Sinus imaging (CT scan/X-ray)	123 (12%)	35 (28%)
GI testing (endoscopy/barium swallow, pH testing)	106 (10%)	34 (32%)
Bronchoscopy	46 (4%)	16 (35%)
Don’t know/none of the above	345 (33%)	NA

CT, computed tomography. GI, gastrointestinal. NA, not applicable. RCC, refractory chronic cough. UCC, unexplained chronic cough.

### Treatments for RCC/UCC

Currently in Canada there are no treatments specifically approved for chronic cough, but a number of therapies are prescribed off-label. Treatments currently prescribed to survey respondents to address RCC/UCC are shown in Table 4. However, 32% of respondents were not prescribed any current treatment for their cough.

**Table 4:** Treatments prescribed/recommended for RCC/UCC^a^. Patients may have been prescribed more than one treatment.

Treatment	Ever prescribed n (%)	Currently prescribed/recommended among those who recall n (%)	Mean years receiving current treatment^b^	Reduced or stopped medication without informing HCP^c^ n (%)
N	1,046	864	864	864
Not currently prescribed any treatment	NA	276 (32%)	NA	NA
Cough suppressant	465 (44%)	159 (18%)	1.7	42 (26%)
Cough drops/syrup	331 (32%)	121 (14%)	3.3	35 (29%)
Antihistamines/allergy relief medications	273 (26%)	139 (16%)	7.3	36 (26%)
Nasal steroids	240 (23%)	131 (15%)	3.4	30 (23%)
Inhaled steroids	237 (23%)	148 (17%)	8.5	33 (23%)
Expectorant	180 (17%)	48 (6%)	2.6	28 (59%)
Decongestant	163 (16%)	51 (6%)	4.1	12 (24%)
Antibiotics	142 (14%)	36 (4%)	2.8	8 (22%)
Beta-agonists	112 (11%)	87 (10%)	9.5	17 (20%)
Proton pump inhibitors	107 (10%)	51 (6%)	5.8	11 (22%)
Oral steroids	86 (8%)	24 (3%)	4.4	9 (37%)
Codeine	72 (7%)	13 (2%)	2.5	6 (46%)
H2 blocker	56 (5%)	20 (2%)	4.9	9 (45%)
Sleeping aid medication	52 (5%)	23 (2%)	4.0	4 (17%)
Neuromodulators (e.g. gabapentin, pregabalin, amitriptyline)	51 (5%)	20 (2%)	2.1	5 (25%)
Antidepressants	42 (4%)	23 (3%)	7.4	2 (8%)
Speech and language therapy	16 (2%)	5 (1%)	0.2	0
Morphine	7 (1%)	2 (<1%)	3.0	0
Can’t remember the name of the treatment	182 (17%)	NA	NA	NA

^a^See S1 Methods Section D for list of treatments and examples provided to respondents

^b^In response to the question: “For how long have you been receiving your currently prescribed/recommended treatment for chronic cough?” (among patients with a current prescribed/recommended treatment)

^c^Response of “all of the time” or “often” to the question: “Do you ever reduce or stop taking one of your prescribed/recommended cough medications without informing your main healthcare professional?” (among patients with a current prescribed/recommended treatment)

HCP, healthcare professional. NA, not applicable. ND, not determined due to small sample size. RCC, refractory chronic cough. UCC, unexplained chronic cough.

The treatment most frequently ever prescribed (44%) or currently prescribed/recommended (18%) for RCC/UCC was cough suppressants. Other common treatments included cough drops/syrup (32% ever prescribed, 14% currently prescribed), antihistamines/allergy relief medications (26% ever prescribed, 16% currently prescribed), inhaled steroids (23% ever prescribed, 17% currently prescribed, and nasal steroids (23% ever prescribed, 15% currently prescribed). (Table 4). Approximately 20% to 30% of respondents reported stopping taking a cough medication often or all the time for most chronic cough treatments, although some treatments showed a much higher proportion of respondents who stopped treatment (expectorants 59%, codeine 46%, and H2 blockers 45%) and others showed a lower proportion (antidepressants 8%, sleeping aid medication 18%) (Table 4). The most common reasons for reducing or stopping currently prescribed/recommended cough treatments were “I forget to take my medication” and “I don’t feel like it is working” (32% each), followed by “I don’t think I need to take the medication as frequently as prescribed” (29%), “I prefer not to take medication” (21%), and “I control my symptoms with non-prescription/over-the-counter (OTC) treatments” (16%).

In addition to frequent use of prescription medications, 77% of respondents reported purchasing non-prescription/OTC treatments to manage their RCC/UCC, and 50% reported trying home remedies to treat cough.

### Satisfaction with RCC/UCC management

**Fig 2:**
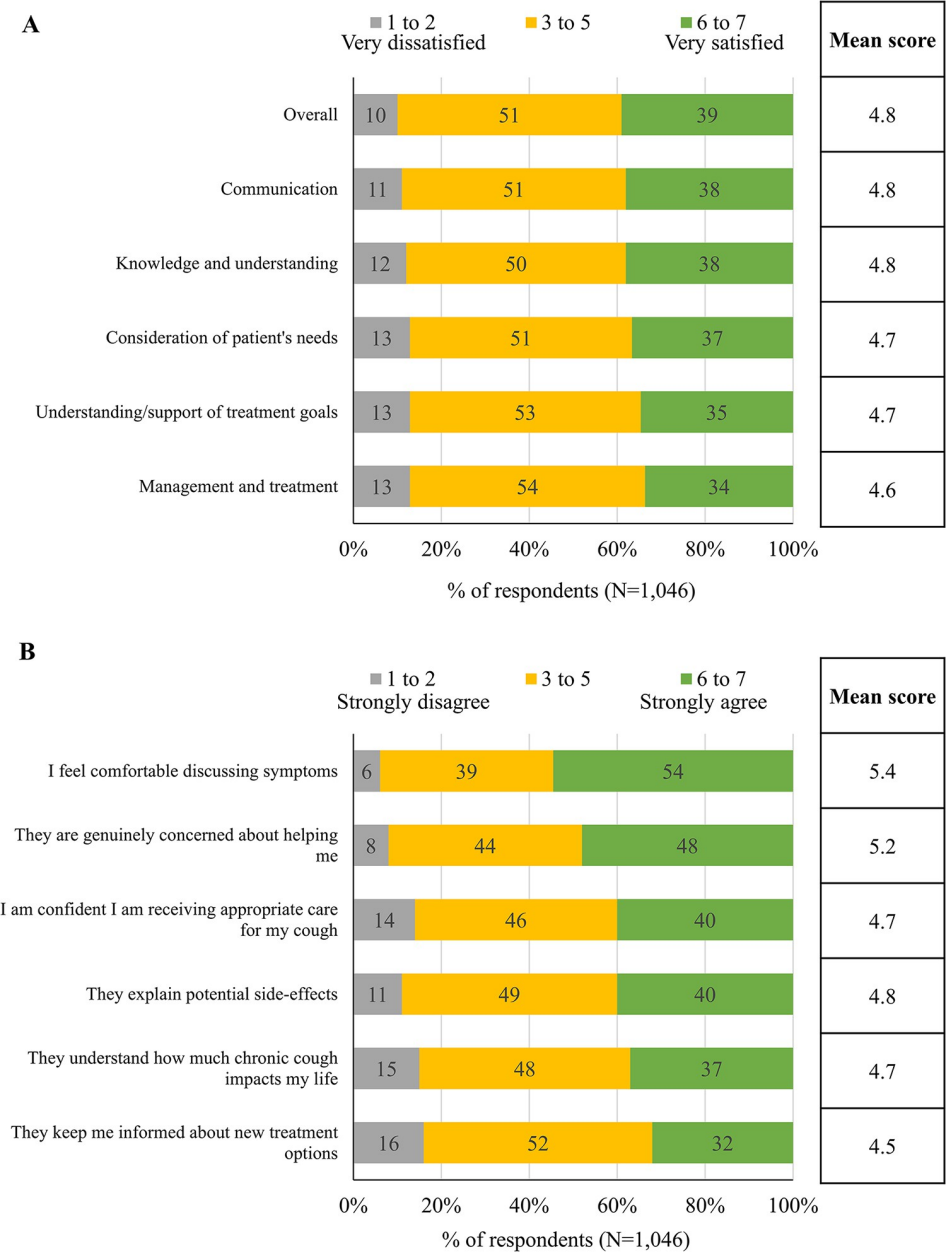
Respondents’ ratings of (A) satisfaction with HCP and (B) agreement with statements about HCP. For (A), respondents were asked “Thinking about the healthcare professional who has MAIN responsibility for your chronic cough treatment and management, overall how satisfied are you with them on the following factors?” (7-point scale where 1 = very dissatisfied and 7 = very satisfied). For (B), respondents were asked “Based on your experience with your healthcare professional who has MAIN responsibility for your chronic cough, to what extent do you agree with the following statements with regards to your chronic cough?” (7-point scale where 1 = strongly disagree and 7 = strongly agree).

Overall, respondents were moderately satisfied with the HCP who had the main responsibility for managing their cough (mean of 4.8 on a 7-point scale of 1 = very dissatisfied to 7 = very satisfied). Similar mean scores were reported for specific aspects of satisfaction, including communication about chronic cough and its treatment and level of knowledge and understanding of chronic cough (Fig 2A). Most respondents (70%) were satisfied with the frequency with which they saw the HCP primarily responsible for managing their RCC/UCC, but 28% reported they did not see the HCP often enough, and 2% felt they saw the HCP too much.

Respondents also expressed moderate agreement with statements concerning their HCPs, such as “I feel comfortable discussing symptoms” (mean of 5.4 on a 7-point scale of 1 = strongly disagree to 7 = strongly agree) and “They explain potential side-effects” (mean of 4.7). The lowest level of agreement was for the statement “They keep me informed about new treatment options” (mean of 4.5) (Fig 2B).

A substantial proportion of patients reported concerns about their treatment’s effectiveness and side-effects (Table 5). There were only 4 agents; expectorants, antibiotics, neuromodulators, and codeine, for which at least half of respondents strongly agreed (6 or 7 on a 7-point scale) with the statement “My treatment is effective at treating my cough symptoms.” For the most commonly-used agents, including cough suppressants, inhaled steroids, antihistamines/allergy relief medication, nasal steroids, and cough/drops/syrups (more than 100 patients), only 21% to 33% of respondents would recommend the treatment to another patient with chronic cough. Worries about short-term and long-term effects of medications were common, particularly for neuromodulators, expectorants, and antibiotics (Table 5). Patients on neuromodulators were the most likely to express concern regarding both the short-term (50%) and long-term effects (60%) of their medication, despite similar proportions acknowledging satisfaction with treatment (60%).

**Table 5:** Respondents’ satisfaction with current treatment. Data are reported as percentages based on the number of patients receiving the specified current treatment who reported a 6 or 7 for that question divided by the total number of patients on the specified treatment.

	Current treatment n	High level of agreement (6 or 7 on a 7-point scale)^a^				
Treatment		I am very satisfied with my treatment’s control of my cough	My treatment is effective at treating my cough symptoms	I would recommend my cough treatment to another cough patient	I am worried about short-term side effects of my cough treatment	I worry about the long-term side effects of my cough treatment
Cough suppressant	159	43%	41%	33%	33%	29%
Inhaled steroids	148	44%	43%	30%	21%	33%
Antihistamines/allergy relief medications	139	35%	29%	21%	14%	27%
Nasal steroids	131	29%	28%	25%	27%	38%
Cough drops/syrup	121	41%	42%	31%	18%	25%
Beta-agonists	87	47%	49%	36%	18%	30%
Proton pump inhibitors	51	31%	29%	27%	25%	43%
Decongestant	51	41%	35%	25%	18%	24%
Expectorant	48	71%	56%	56%	48%	42%
Antibiotics	36	53%	53%	44%	42%	42%
Oral steroids	24	33%	25%	29%	25%	42%
Antidepressants	23	17%	13%	13%	13%	17%
Sleeping aid medication	23	30%	17%	35%	26%	39%
Neuromodulators (e.g. gabapentin, pregabalin, amitriptyline)	20	60%	50%	55%	50%	60%
H2 blockers	20	45%	40%	20%	30%	25%
Codeine	13	38%	62%	38%	0	23%
Speech and language therapy	5	60%	40%	40%	20%	0
Morphine	2	0	0	50%	50%	0

^a^On a 7-point scale ranging from 1 = strongly disagree to 7 = strongly agree.

**Fig 3:**
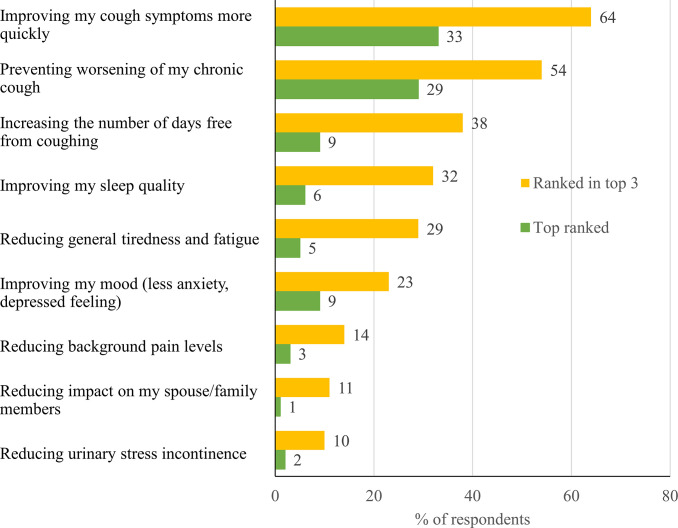
Respondents’ perceptions of the most important benefits of cough treatment. Respondents (N = 1,046) were asked: “Other than a cure for your chronic cough, what are the THREE most important benefits a cough treatment could provide? Please assign rankings (1 to 3) with 1 being the most important, 2 the next most important, etc.” Responses that were ranked in the top 3 by >5% of respondents are shown.

When asked about the most important benefits a cough treatment could provide (other than a cure), the top-ranked benefit was improving cough symptoms more quickly (64% of respondents rated this in the top 3), followed by preventing worsening of my chronic cough (54%) and increasing the number of days free from coughing (38%) (Fig 3).

## Discussion

Our survey of Canadian individuals with current, ongoing RCC/UCC documented a complicated treatment path involving multiple HCPs and leaving more than half of patients on most drugs unsatisfied with the ability of their current treatment to control their cough. Based on the entry criteria used for this study, 3.3% of the screened population and 24.7% of individuals with self-reported current chronic cough had RCC or UCC. Forty-four percent of those respondents did not have a diagnosis of an underlying condition related to their cough or have a serious condition ruled out, and 20% were currently waiting for an appointment with a specialist related to their chronic cough. The frustration associated with the RCC/UCC patient journey is perhaps best summarized by the fact that 34% had at some point chosen to stop seeking further medical attention for their cough because of a lack of success in treating it.

The findings from this survey are consistent with patient experiences in other regions, including the US [9], Europe [7, 21], and Asia [29, 30]. As has been noted in other studies, long durations of chronic cough are common. There appears to be a consensus among chronic cough patients worldwide that underlying etiologies for their cough are often elusive, even after careful testing, and that current treatments are largely ineffective.

For most respondents, chronic cough was managed by a GP, but other HCPs, including specialists, nurses, and pharmacists, were also involved. There were only minimal differences in the perceived usefulness of different practitioners, which may reflect the lack of understanding of RCC/UCC as a disease as well as the lack of useful treatments to improve the condition. In addition to consulting HCPs, substantial proportions of patients utilized internet sources or turned to family or friends to learn more about their condition. This finding suggests that patients had unanswered questions about their chronic cough. Online RCC/UCC groups to provide additional support and education might help fill this gap.

As was found in the study reported here, respondents to other surveys are generally satisfied with the efforts of their HCPs. As one patient commented, “I just think it’s poorly understood, and it’s not their [the HCPs] fault; it’s just the way it is” [31]. However, in our survey a large proportion of patients did not receive common diagnostic tests; this finding may have been influenced by patient recall (33% answered “don’t know/none of the above” to questions about diagnostic tests). Recent surveys suggest that better education for HCPs on chronic cough may improve management and adherence to guidelines [10–14].

Although educational efforts are an important component of improved care for patients with chronic cough, it is likely that novel management options are required to fully meet the needs of these patients. Commonly prescribed therapies such as cough suppressants, inhaled steroids, and decongestants had poorly rated effectiveness by respondents. More effectively rated therapies such as neuromodulators and opiates were noted to provoke concern for short- and long-term side effects. Perhaps this is the reason why these were less commonly used despite being recommended by current American College of Chest Physicians/European Respiratory Society guidelines [3, 15]. Other potential reasons for the low use of recommended therapies include concern with prescribing off-label drugs. Speech therapy, another recommended therapy, appeared to be under-utilized in Canadian patients with RCC/UCC. This therapeutic modality is not readily available in some areas; lack of physician awareness of the effectiveness of this therapy may also be an issue. Respondents in our study listed the top-ranked benefit of a cough treatment as improving cough symptoms more quickly, preventing worsening of the cough, and increasing the number of days free from coughing. It is possible these goals may be achieved by new therapies on the horizon, including antagonists of P2X3 receptors, which are believed to be involved in cough reflex activation [34] and lack the sedative effects of centrally acting neuromodulators currently used off-label for the management of cough. One P2X3 receptor antagonist, gefapixant, has reported successful phase 3 outcomes [35, 36] and received regulatory approval for RCC/UCC in Japan, Switzerland, and the European Commission. Compared with placebo, gefapixant 45 mg showed reductions in 24-hour cough frequency by week 4. The drug was well tolerated but side effects related to taste were common [35].

Limitations of our study include possible sampling bias toward respondents who have greater interest in or impact from chronic cough and, as mentioned above, potential self-report and recall biases, particularly with respect to diagnostic tests. The exclusion of individuals who are current smokers, who represented 34% of Canadians between 45 and 85 years at age with chronic cough in the Canadian Longitudinal Study on Aging [1], and of other respondents with specified chronic cough etiologies is both a limitation and a study strength, as it provides insights into the burden of chronic cough in individuals who are likely to have RCC or UCC. The study could not verify that patients underwent a full suite of diagnostic tests (and self-reports suggest that many did not), possibly resulting in overestimation of UCC in the survey respondents. In addition, the study did not exclude individuals with GERD and asthma under the assumption that the patient’s cough persisted even after appropriate management of these known conditions and therefore represented RCC. However, given the survey design, appropriate treatment or medication adherence in these patients could not be verified, and it is therefore possible that improved management of these underlying conditions may have resulted in cough resolution. Similarly, failed therapies for conditions that may have contributed to RCC were not documented. The study was focused on patients with current, ongoing RCC/UCC so patients who received successful treatment with other therapies were excluded. This may have obscured the effect of potentially successful therapies, such as speech therapy [37–39].

In conclusion, individuals with RCC/UCC show low to moderate satisfaction with the management of their condition and a substantial proportion find their current treatment to be ineffective. Additional education for HCPs on guidelines and best practices for the diagnosis and treatment of chronic cough may help patients improve underlying disorders for better cough control or access existing therapies with some benefit. However, for the substantial proportion of patients with RCC or UCC, improved satisfaction with management may require new treatment options.

## Supporting information

S1 Methods Screening questionnaire and survey. (DOCX)
