# Supplementary material for: Patient satisfaction with the management of refractory and unexplained chronic cough in Canada: Results from a national survey
Source: PLoS One. 2024 Aug 1;19(8):e0308275. doi: 10.1371/journal.pone.0308275 (PMC11293633; doi:10.1371/journal.pone.0308275)
Supplement: S1 Methods — (DOCX) [file pone.0308275.s001.docx]

**Supporting information**

**S1 Methods. Screening Questionnaire and Survey**

Thank you for participating in this online survey on Chronic Cough. Your opinions are very important to us. Please fill out the following survey that should take about **30 minutes** to complete.

Remember, all of the information you provide is kept strictly confidential and used for market research purposes only. Your specific information will never be given to anyone.

This survey is being conducted by Leger, an independent market research agency working within specific Market Research Codes of Conduct, on behalf of a pharmaceutical company. You have the right to refuse to answer any question, or withdraw from participation at any time.

By selecting “I Consent” below, you certify that you are eighteen (18) years old or older. you acknowledge and confirm you understand that all survey materials are proprietary to the survey owner and must not be used for any other purpose than participation in the survey or disclosed to any third party without the express written permission of the survey owner..

□ I consent.

□ I do not consent. [Terminate]

If you have questions, concerns, or complaints, please contact:

Dima Ostrikov at dostrikov@leger360.com.

**SCREENER**

**PN: PLEASE ALLOW TO COMPLETE THE SCREENER AND THEN TERMINATE IF A RESPONDENT DOESN’T QUALIFY**

S1. How **old** are you?

|  | Years old | **TERMINATE IF <18 YEARS** |
| --- | --- | --- |

S2. What is your **gender**?

| Female | 1 |
| --- | --- |
| Male | 2 |
| Other | 3 |
| I prefer not to answer | 99 |

S3. Please specify the **province** the province in which you live.

|  |  | **QUOTAS** |
| --- | --- | --- |
| British Columbia | 1 | **150-160** |
| Alberta | 2 | **160-170** |
| Saskatchewan | 3 |  |
| Manitoba | 4 |  |
| Ontario | 5 | **360-370** |
| Quebec | 6 | **275-285** |
| New Brunswick | 7 | **50-60** |
| Nova Scotia | 8 |  |
| Prince Edward Island | 9 |  |
| Newfoundland | 10 |  |
| Territories | 11 | **TERMINATE** |
|  |  | **N = 1,000** |

S4. In general, how would you **describe your current health**?

Please select a rating below, from poor health to excellent health

| **1** | **2** | **3** | **4** | **5** |
| --- | --- | --- | --- | --- |
| Poor | Fair | Good | Very good | Excellent |

S5a. Have you **ever smoked** cigarettes, marijuana or other tobacco products, including electronic cigarettes (e-cigarettes) or other vaping devices? *Please select one only*

| 1 | Yes, I smoke currently | **TERMINATE** |
| --- | --- | --- |
| 2 | Yes, but I quit | **ASK S5ai** |
| 3 | No, I have never smoked | **CONTINUE TO S7** |
| 4 | Prefer not to say | **TERMINATE** |

**PN: ASK IF CODE 2 SELECTED AT S5a**

S5ai. Which of the following tobacco products did you use to smoke typically? *Please select all that apply.*

| 1 | Cigarettes | 1 |
| --- | --- | --- |
| 2 | Electronic cigarettes (e-cigarettes) or other vaping devices | 2 |
| 3 | Marijuana | 3 |
| 4 | Other tobacco products | 4 |

**ASK IF CODE 2 AT S5a**

S5b. When did you quit smoking?

| 1 | Less than 1 year ago | **TERMINATE** |
| --- | --- | --- |
| 2 | 1 year to less than 2 years ago |  |
| 3 | 2 years to less than 3 years ago |  |
| 4 | 3-4 years ago |  |
| 5 | 5-10 years ago |  |
| 6 | Over 10 years ago |  |

**PN: ASK IF CODE 1 SELECTED AT S5ai**

S5c. Approximately, how many packs of cigarettes, did you use to smoke per year?

|  | # packs per year | **TERMINATE IF ≥20 PACKS** |
| --- | --- | --- |

S6. Have you **experienced a cough** on most days for the **past 8 weeks or longer** despite taking treatment for the cough or taking treatment for the medical condition that may be related to the cough?

| 1 | ⭘ | Yes |  |
| --- | --- | --- | --- |
| 2 | ⭘ | No | **TERMINATE** |

S7. Have you ever been diagnosed with any of the following medical conditions? *Please select all that apply*.

| 1 |  | Idiopathic pulmonary fibrosis (IPF) | **TERMINATE** |
| --- | --- | --- | --- |
| 2 |  | Chronic obstructive pulmonary disease (COPD) | **TERMINATE** |
| 3 |  | Lung cancer | **TERMINATE** |
| 99 |  | None of the above |  |

S8. Have you ever been diagnosed with COVID-19? *Please select one.*

| Yes, I have been diagnosed with COVID-19 | 1 | **TERMINATE** |
| --- | --- | --- |
| No, I have not been diagnosed with COVID-19 and have not experienced symptoms associated with COVID-19 | 2 | **TERMINATE** |
| I prefer not to answer | 99 | **TERMINATE** |

**MONITOR TERMINATES AT THIS QUESTION**

S9. Are you **currently** participating in any clinical trial(s) sponsored by a pharmaceutical company?

| 1 | ⭘ | Yes | **TERMINATE** |
| --- | --- | --- | --- |
| 2 | ⭘ | No |  |

**SHOW ON SEPARATE PAGE:
ADVERSE EVENT STATEMENT: SHOW ON SEPARATE SCREEN**

You are about to enter a market research interview. We are now being asked to pass on to our clients details of adverse events that are raised during the course of market research interviews. Although this is an on-line market research interview and how you respond will, of course, be treated in confidence, should you raise an adverse event or a product complaint, we will need to report this, even if it has already been reported by you directly to the company or the regulatory authority.

In such a situation you will be contacted to ask whether you are willing to waive the confidentiality given to you under the Market Research Codes of Conduct specifically in relation to that adverse event. Everything else you contribute during the course of the interview will continue to remain confidential.

If you agree to be contacted, then your name and contact details will be forwarded to the sponsor’s Product Safety Department for the sole purpose of follow-up to the report(s) of an adverse event.

If you do not agree to be contacted, then we will forward the report(s) of any adverse event to the sponsor’s Product Safety Department, but you (and any patient) will remain completely anonymous.

**Please read the statements below and click on your answer:**

- I would like to proceed and give permission for my contact details to be passed on to the Drug Safety department of the company if an adverse event/ product complaint is mentioned by me during the survey **(PROCEED WITH MARKET RESEARCH)**
- I would like to proceed but do not wish for my contact details to be passed on to the Drug Safety department of the company if an adverse event/ product complaint is mentioned by me during the survey **(PROCEED WITH MARKET RESEARCH)**

I don’t want to proceed and wish to end the interview here **(THANK YOU AND CLOSE. DO NOT PROCEED WITH MARKET RESEARCH)**

**SECTION A: GENERAL HEALTH [SECTION HEADER – DO NOT SHOW]**

A1. Which of the following conditions have you **ever been** diagnosed with?

A2. Of those, which of these conditions are you **currently** diagnosed with?

*Please select all that apply*

|  | a. Conditions I have EVER been diagnosed with | b. Conditions I am CURRENTLY diagnosed with |
| --- | --- | --- |
| 1. Myocardial infarction (heart attack) | o | o |
| 2. Congestive heart failure | o | o |
| 3. Peripheral arterial / vascular disease (PAD / PVD) | o | o |
| 4. Cerebrovascular disease (e.g. stroke, vascular dementia) | o | o |
| 5. Hemiplegia or paraplegia (paralysis) | o | o |
| 6. Dementia | o | o |
| 7. Chronic pulmonary disease (chronic bronchitis and/or emphysema) | o | o |
| 8. Rheumatologic disease (e.g. osteoarthritis, rheumatoid arthritis) | o | o |
| 9. Peptic Ulcer Disease (stomach ulcers) | o | o |
| 10. Diabetes (Type I or Type II) **without** chronic complications (e.g. diabetics foot, loss of sight) | o | o |
| 11. Diabetes (Type I or Type II) **with** chronic complications (e.g. diabetics foot and loss of sight) | o | o |
| 12. Obesity | o | o |
| 13. Kidney disease | o | o |
| 14. Any cancer, including lymphoma | o | o |
| 15. Metastatic solid tumor | o | o |
| 16. Mild liver disease | o | o |
| 17. Moderate or severe liver disease | o | o |
| 18. Thyroid disease (overactive / underactive thyroid) | o | o |
| 19. HIV / AIDS | o | o |
| 20. Bladder problems (e.g. incontinence and chronic pain) | o | o |
| 21. Chronic pain (e.g. neuralgia, neuropathy) | o | o |
| 22. Healthcare associated infections (HCAI) | o | o |
| 23. Other (specify) __________ | o | o |
| 24. None of the above | ⭘ | ⭘ |

[EQ5D-5L - **DO NOT SHOW**]

A3. Under each heading, please select the ONE box that best describes your health TODAY.

1. **Mobility**

| I have no problems in walking about | 🞎 |
| --- | --- |
| I have slight problems in walking about | 🞎 |
| I have moderate problems in walking about | 🞎 |
| I have severe problems in walking about | 🞎 |
| I am confined to bed | 🞎 |

1. **Self-Care**

| I have no problems washing or dressing myself | 🞎 |
| --- | --- |
| I have slight problems washing or dressing myself | 🞎 |
| I have moderate problems washing or dressing myself | 🞎 |
| I have severe problems washing or dressing myself | 🞎 |
| I am unable to wash or dress myself | 🞎 |

1. **Usual Activities** *(e.g. work, study, housework, family or leisure activities)*

| I have no problems doing my usual activities | 🞎 |
| --- | --- |
| I have slight problems doing my usual activities | 🞎 |
| I have moderate problems doing my usual activities | 🞎 |
| I have severe problems doing my usual activities | 🞎 |
| I am unable to do my usual activities | 🞎 |

1. **Pain / Discomfort**

| I have no pain or discomfort | 🞎 |
| --- | --- |
| I have slight pain or discomfort | 🞎 |
| I have moderate pain or discomfort | 🞎 |
| I have severe pain or discomfort | 🞎 |
| I have extreme pain or discomfort | 🞎 |

1. **Anxiety / Depression**

| I am not anxious or depressed | 🞎 |
| --- | --- |
| I am slightly anxious or depressed | 🞎 |
| I am moderately anxious or depressed | 🞎 |
| I am severely anxious or depressed | 🞎 |
| I am extremely anxious or depressed | 🞎 |

[Q5- EQ5D-VAS- **DO NOT SHOW**]


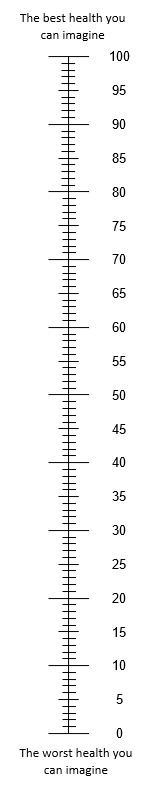
A4. We would like to know how good or bad your health is TODAY.

Please select a number on the scale that best describes your health.

This scale is numbered from 0 to 100.

- 100 means the best health you can imagine.
- 0 means the worst health you can imagine.

**SECTION B: EXPERIENCE AND IMPACT OF CHRONIC COUGH ON QUALITY OF LIFE [SECTION HEADER – DO NOT SHOW]**

B1. For how long have you experienced a daily or almost daily cough?

***Please write your best estimate, in months or years.***

| 1 | ⭘ | ______ months **[SKIP TO B4 IF LESS THAN 12 MONTHS]** |
| --- | --- | --- |
| 2 | ⭘ | ______ years |
| 3 | ⭘ | Don’t know **[SKIP TO B4]** |

**[PN: ASK ONLY IF 12 MONTHS OR MORE AT B1]**

**B2.** Is your chronic cough more severe at a certain time or season of the year?

***Please select one answer.***

| 1 | ⭘ | Yes |
| --- | --- | --- |
| 2 | ⭘ | No **[SKIP GO TO B4]** |

**[PN: ASK ONLY IF 12 MONTHS OR MORE AT B1 AND YES AT B2]**

**B3.** What time of year is your chronic cough most severe?

***Please select one answer.***

| 1 | ⭘ | Winter (December, January, February) |
| --- | --- | --- |
| 2 | ⭘ | Spring (March, April, May) |
| 3 | ⭘ | Summer (June, July, August) |
| 4 | ⭘ | Autumn (September, October, November) |

[COUGH-VAS - **DO NOT SHOW**]

**B4.** How severe was your cough today?

Please rate the severity of your cough by Please select a number on the scale that best describes severe was your cough **today**.

Extremely Severe Cough

(100mm)

No Cough

(0mm)

**PN: SCALE OF 100 mm WITH NO COUGH AT 0 MM AND EXTREMELY SEVERE COUGH AT 100 MM**

[LEICESTER COUGH QUESTIONNAIRE - DO NOT SHOW]

**B5.** This section is designed to assess the impact of cough on various aspects of your life. Read each question carefully and answer by SELECTING the response that best applies to you. Please answer ALL questions, as honestly as you can.

1. In the last 2 weeks, have you had chest or stomach pains as a result of your cough?

| 1 | 2 | 3 | 4 | 5 | 6 | 7 |
| --- | --- | --- | --- | --- | --- | --- |
| All of the time | Most of  the time | A good bit  of the time | Some of  the time | A little of  the time | Hardly any of the time | None of  the time |

2. In the last 2 weeks, have you been bothered by sputum (phlegm) production when you cough?

| 1 | 2 | 3 | 4 | 5 | 6 | 7 |
| --- | --- | --- | --- | --- | --- | --- |
| Every time | Most times | Several times | Sometimes | Occasionally | Rarely | Never |

3. In the last 2 weeks, have you been tired because of your cough?

| 1 | 2 | 3 | 4 | 5 | 6 | 7 |
| --- | --- | --- | --- | --- | --- | --- |
| All of  the time | Most of  the time | A good bit  of the time | Some of  the time | A little of  the time | Hardly any of the time | None of  the time |

4. In the last 2 weeks, have you felt in control of your cough?

| 1 | 2 | 3 | 4 | 5 | 6 | 7 |
| --- | --- | --- | --- | --- | --- | --- |
| None of  the time | Hardly any of  the time | A little of the time | Some of  the time | A good bit of  the time | Most of the time | All of  the time |

5. How often during the last 2 weeks have you felt embarrassed by your coughing?

| 1 | 2 | 3 | 4 | 5 | 6 | 7 |
| --- | --- | --- | --- | --- | --- | --- |
| All of  the time | Most of  the time | A good bit  of the time | Some of  the time | A little of  the time | Hardly any of the time | None of  the time |

6. In the last 2 weeks, my cough made me feel anxious

| 1 | 2 | 3 | 4 | 5 | 6 | 7 |
| --- | --- | --- | --- | --- | --- | --- |
| All of the time | Most of  the time | A good bit  of the time | Some of  the time | A little of  the time | Hardly any of the time | None of  the time |

7. In the last 2 weeks, my cough has interfered with my job or other daily tasks

| 1 | 2 | 3 | 4 | 5 | 6 | 7 |
| --- | --- | --- | --- | --- | --- | --- |
| All of the time | Most of  the time | A good bit  of the time | Some of  the time | A little of  the time | Hardly any of the time | None of  the time |

8. In the last 2 weeks, I felt that my cough interfered with the overall enjoyment of my life

| 1 | 2 | 3 | 4 | 5 | 6 | 7 |
| --- | --- | --- | --- | --- | --- | --- |
| All of the time | Most of  the time | A good bit  of the time | Some of  the time | A little of  the time | Hardly any of the time | None of  the time |

9. In the last 2 weeks, exposure to paint or fumes has made me cough

| 1 | 2 | 3 | 4 | 5 | 6 | 7 |
| --- | --- | --- | --- | --- | --- | --- |
| All of the time | Most of  the time | A good bit  of the time | Some of  the time | A little of  the time | Hardly any of the time | None of  the time |

10. In the last 2 weeks, has your cough disturbed your sleep?

| 1 | 2 | 3 | 4 | 5 | 6 | 7 |
| --- | --- | --- | --- | --- | --- | --- |
| All of the time | Most of  the time | A good bit  of the time | Some of  the time | A little of  the time | Hardly any of the time | None of  the time |

11. In the last 2 weeks, how many times a day have you had coughing bouts?

| 1 | 2 | 3 | 4 | 5 | 6 | 7 |
| --- | --- | --- | --- | --- | --- | --- |
| All of the time (continuously) | Most times during the day | Several times during the day | Sometimes during the day | Occasionally during the day | Rarely | None |

12. In the last 2 weeks, my cough has made me feel frustrated

| 1 | 2 | 3 | 4 | 5 | 6 | 7 |
| --- | --- | --- | --- | --- | --- | --- |
| All of the time | Most of  the time | A good bit  of the time | Some of  the time | A little of  the time | Hardly any of the time | None of  the time |

13. In the last 2 weeks, my cough has made me feel fed up

| 1 | 2 | 3 | 4 | 5 | 6 | 7 |
| --- | --- | --- | --- | --- | --- | --- |
| All of the time | Most of  the time | A good bit  of the time | Some of  the time | A little of  the time | Hardly any of the time | None of  the time |

14. In the last 2 weeks, have you suffered from a hoarse voice as a result of your cough?

| 1 | 2 | 3 | 4 | 5 | 6 | 7 |
| --- | --- | --- | --- | --- | --- | --- |
| All of the time | Most of  the time | A good bit  of the time | Some of  the time | A little of  the time | Hardly any of the time | None of  the time |

15. In the last 2 weeks, have you had a lot of energy?

| 1 | 2 | 3 | 4 | 5 | 6 | 7 |
| --- | --- | --- | --- | --- | --- | --- |
| None of  the time | Hardly any of  the time | A little of the time | Some of  the time | A good bit of  the time | Most of the time | All of  the time |

16. In the last 2 weeks, have you worried that your cough may indicate serious illness?

| 1 | 2 | 3 | 4 | 5 | 6 | 7 |
| --- | --- | --- | --- | --- | --- | --- |
| All of the time | Most of  the time | A good bit  of the time | Some of  the time | A little of  the time | Hardly any of the time | None of  the time |

17. In the last 2 weeks, have you been concerned that other people think something is wrong with you, because of your cough?

| 1 | 2 | 3 | 4 | 5 | 6 | 7 |
| --- | --- | --- | --- | --- | --- | --- |
| All of the time | Most of  the time | A good bit  of the time | Some of  the time | A little of  the time | Hardly any of the time | None of  the time |

18. In the last 2 weeks, my cough has interrupted conversation or telephone calls

| 1 | 2 | 3 | 4 | 5 | 6 | 7 |
| --- | --- | --- | --- | --- | --- | --- |
| Every time | Most times | A good bit  of the time | Some of  the time | A little of  the time | Hardly any of the time | None of  the time |

19. In the last 2 weeks, I feel that my cough has annoyed my partner, family, or friends

| 1 | 2 | 3 | 4 | 5 | 6 | 7 |
| --- | --- | --- | --- | --- | --- | --- |
| Every time I cough | Most times when I cough | Several times when I cough | Sometimes when I cough | Occasionally when I cough | Rarely | Never |

B6. In the last 2 weeks, have you been bothered by urinary incontinence when you cough?

| 1 | 2 | 3 | 4 | 5 | 6 | 7 |
| --- | --- | --- | --- | --- | --- | --- |
| Every time | Most times | Several times | Sometimes | Occasionally | Rarely | Never |

**SECTION C: REFERRAL PATHWAY & HCP-PATIENT RELATIONSHIPS [SECTION HEADER – DO NOT SHOW]**

C1. What prompted you to first talk to a doctor or nurse about your cough?

***Please select one answer.***

| 1 | ⭘ | I was seeing my doctor about something else and they noticed my cough |
| --- | --- | --- |
| 2 | ⭘ | I decided to talk to a doctor myself because my cough was worrying me |
| 3 | ⭘ | I was encouraged to talk to a doctor by family and / or friends to discuss my cough |
| 4 | ⭘ | I was encouraged to talk to a doctor by my caregiver to discuss my cough |
| 5 | ⭘ | Another reason (please specify _______________________________) |

C2. How long after your cough started did it take before you first spoke to a doctor or nurse about it? ***Please write your best estimate, in weeks or months.***

| 1 | ⭘ | It took ______ weeks |
| --- | --- | --- |
| 2 | ⭘ | It took ______ months |
| 3 | ⭘ | Don’t know |

C3a. Have you ever been informed or diagnosed with a condition as the reason for your cough?

***Please select one answer.***

| 1 | ⭘ | Yes |
| --- | --- | --- |
| 2 | ⭘ | No **[SKIP TO C4]** |
| 3 | ⭘ | Don’t know **[SKIP TO C4]** |

**PN: ASK IF RECEIVED A DIAGNOSIS – CODE 1 AT C3a**

C3b. To the best of your knowledge, how many healthcare professionals (e.g. doctors, nurses, respiratory therapists, pharmacists, etc.) did you see before being diagnosed with a condition as the reason for your cough or receiving confirmation of no serious underlying cause?

***Please write your best estimate.***

| ________ number of healthcare professionals seen  ________ number of visits in total to healthcare professionals |
| --- |

C4. Following your initial visit to a healthcare professional (doctor, nurse, respiratory therapist, pharmacist, etc.), how long was it before you received a diagnosis of the underlying cause for your cough or confirmation that it is not due to a serious underlying condition?

***Please select one answer.***

| 🞎 | Within first 3 months |
| --- | --- |
| 🞎 | Within a year |
| 🞎 | Greater than a year |
| 🞎 | Still no underlying diagnosis |

**PN: ASK IF CODE 1 AT C4**

C5. In your own opinion, why do you think there was a delay in receiving an explanation for your chronic cough?

*Please select all that apply*

| 🞎 | The doctor told me to monitor the symptoms to see if they improved |
| --- | --- |
| 🞎 | I had to wait to be referred to a specialist who could provide this explanation |
| 🞎 | I had to wait for additional tests conducted so that my doctor could provide this explanation |
| 🞎 | I was first diagnosed with another condition |
| 🞎 | Other |
| ⭘ | Don’t know |

C6a. Which, if any, of these health care professionals have you ever seen to evaluate your chronic cough since experiencing symptoms?

C6b. And how many times have you seen these health care professionals to evaluate your chronic cough in the last 3 months?

***Please select all that apply and enter a number for each.***

|  |  | C6a | C6b |
| --- | --- | --- | --- |
|  |  | EVER seen to  evaluate cough | Number of times seen to evaluate cough IN LAST 3 MONTHS |
| 1 | GP (General physician) | 🞎 | _______ |
| 2 | Respirologist / Pulmonologist / Lung Specialist | 🞎 | _______ |
| 3 | Allergist | 🞎 | _______ |
| 4 | Ear, nose and throat (ENT; otolaryngologist) | 🞎 | _______ |
| 5 | Head and neck surgeon | 🞎 | _______ |
| 6 | Gastroenterologist | 🞎 | _______ |
| 7 | Urologist | 🞎 | _______ |
| 8 | Nurse | 🞎 | _______ |
| 9 | Speech and Language Therapist | 🞎 | _______ |
| 10 | Physiotherapist | 🞎 | _______ |
| 11 | Pharmacist | 🞎 | _______ |
| 12 | Respiratory therapist | 🞎 | _______ |
| 13 | Other (please specify) ______________ | 🞎 | _______ |

C6c. How useful did you find each of the following health care professionals as sources of information for your chronic cough?

Please rate on a scale of 1 to 7, where 1 is ‘not useful at all’ and 7 is ‘extremely useful’.

**PN: SHOW ONLY THOSE SELECTED AT C6a**

| 1 | 🞎 | GP (General physician) |
| --- | --- | --- |
| 2 | 🞎 | Respirologist / Pulmonologist / Lung Specialist |
| 3 | 🞎 | Allergist |
| 4 | 🞎 | Ear, nose and throat (ENT; otolaryngologist) |
| 5 | 🞎 | Head and neck surgeon |
| 6 | 🞎 | Gastroenterologist |
| 7 | 🞎 | Urologist |
| 8 | 🞎 | Nurse |
| 9 | 🞎 | Speech and Language Therapist |
| 10 | 🞎 | Physiotherapist |
| 11 | 🞎 | Pharmacist |
| 12 | 🞎 | Respiratory therapist |
| 13 | 🞎 | Other (please specify) ______________ |

C6d. Other than physicians, where have you gone for help to learn more about chronic cough and/or your condition? ***Please select all that apply or ‘None of the above’***

| 1 | 🞎 | Patient Associations (including their websites) |
| --- | --- | --- |
| 2 | 🞎 | General online internet searches |
| 3 | 🞎 | Family or friends |
| 4 | 🞎 | Other chronic cough sufferers |
| 5 | 🞎 | Naturopath |
| 6 | 🞎 | Homeopath |
| 7 | 🞎 | Other (please specify) _______ |
| 8 | ⭘ | None of the above |

**PN: SKIP IF CODE 8 SELECTED AT C6d**

C6e. Thinking again about sources of information for your chronic cough, how useful did you find each of the following sources?

**Please rate on a scale of 1 to 7, where 1 is ‘not useful at all’ and 7 is ‘extremely useful’.**

**PN: SHOW ONLY THOSE SELECTED AT C6d**

| 1 | 🞎 | Patient Associations (including their websites) |
| --- | --- | --- |
| 2 | 🞎 | General online internet searches |
| 3 | 🞎 | Family or friends |
| 4 | 🞎 | Other chronic cough sufferers |
| 5 | 🞎 | Naturopath |
| 6 | 🞎 | Homeopath |
| 7 | 🞎 | Other (please specify) _______ |

C7a. And who do you consider to be the main person responsible for the treatment and management of your cough **at this time**? ***Please select one answer.***

**PN: SHOW ONLY THOSE SELECTED AT C6a**

|  |  | MAIN healthcare professional |
| --- | --- | --- |
| 1 | GP (General physician) | ⭘ |
| 2 | Respirologist / Pulmonologist / Lung Specialist | ⭘ |
| 3 | Allergist | ⭘ |
| 4 | Ear, nose and throat (ENT; otolaryngologist) | ⭘ |
| 5 | Head and neck surgeon | ⭘ |
| 6 | Gastroenterologist | ⭘ |
| 7 | Urologist | ⭘ |
| 8 | Nurse | ⭘ |
| 9 | Speech and Language Therapist | ⭘ |
| 10 | Physiotherapist | ⭘ |
| 11 | Pharmacist | ⭘ |
| 12 | Respiratory therapist | ⭘ |
| 13 | Other (please specify) ______________ | ⭘ |

C7b. Are you currently waiting for an appointment (i.e. have you been referred) to see any of the following?

***Please select all that apply or ‘Neither’***

| 1 | 🞎 | Respirologist / Pulmonologist / Lung Specialist |
| --- | --- | --- |
| 2 | 🞎 | Allergists |
| 3 | 🞎 | Ear, nose and throat (ENT; otolaryngologist) |
| 4 | 🞎 | Gastroenterologist |
| 5 | ⭘ | Neither **[SKIP TO SECTION E]** |

C8. How do you feel about how frequently you see your healthcare professional who has main responsibility for your cough?

***Please select one answer.***

| 1 | ⭘ | It is not enough |
| --- | --- | --- |
| 2 | ⭘ | It is enough |
| 3 | ⭘ | It is too much |

C9. Thinking about the healthcare professional who has **MAIN** responsibility for your chronic cough treatment and management, overall how satisfied are you with them on the following factors:

Please rate each statement below, where 1 (very dissatisfied); to 7 (very satisfied).

|  |  | 1  Very dissatisfied | 2 | 3 | 4 | 5 | 6 | 7  Very satisfied |
| --- | --- | --- | --- | --- | --- | --- | --- | --- |
| a. | **Communication** about your chronic cough and its treatment | ⭘ | ⭘ | ⭘ | ⭘ | ⭘ | ⭘ | ⭘ |
| b. | **Management** **and treatment** of your chronic cough | ⭘ | ⭘ | ⭘ | ⭘ | ⭘ | ⭘ | ⭘ |
| c. | **Understanding and support** of your treatment goals | ⭘ | ⭘ | ⭘ | ⭘ | ⭘ | ⭘ | ⭘ |
| d. | **Consideration of your needs** in designing your treatment goals | ⭘ | ⭘ | ⭘ | ⭘ | ⭘ | ⭘ | ⭘ |
| e. | Their level of **knowledge and understanding** of chronic cough | ⭘ | ⭘ | ⭘ | ⭘ | ⭘ | ⭘ | ⭘ |

C10. Overall, how satisfied are you with your experiences with your healthcare professional who has main responsibility for your chronic cough?

Please rate your overall experience below, where 1 (very dissatisfied); to 7 (very satisfied).

| 1  Very dissatisfied | 2 | 3 | 4 | 5 | 6 | 7  Very satisfied |
| --- | --- | --- | --- | --- | --- | --- |
| ⭘ | ⭘ | ⭘ | ⭘ | ⭘ | ⭘ | ⭘ |

C11. Based on your experience with your healthcare professional who has main responsibility for your chronic cough, to what extent do you agree with the following statements with regards to your chronic cough

**Please rate each statement below, where 1 (strongly disagree); to 7 (strongly disagree)**

|  |  | 1 Strongly disagree | 2 | 3 | 4 | 5 | 6 | 7 Strongly agree |
| --- | --- | --- | --- | --- | --- | --- | --- | --- |
| a | They are genuinely concerned about helping me | ⭘ | ⭘ | ⭘ | ⭘ | ⭘ | ⭘ | ⭘ |
| b | They keep me informed about new treatment options | ⭘ | ⭘ | ⭘ | ⭘ | ⭘ | ⭘ | ⭘ |
| c | They ask me about my symptoms at every appointment | ⭘ | ⭘ | ⭘ | ⭘ | ⭘ | ⭘ | ⭘ |
| d | They understand how much my chronic cough impacts my life | ⭘ | ⭘ | ⭘ | ⭘ | ⭘ | ⭘ | ⭘ |
| e | I feel comfortable discussing my symptoms with them | ⭘ | ⭘ | ⭘ | ⭘ | ⭘ | ⭘ | ⭘ |
| f | There is always time during the consultation to discuss all my symptoms and even other problems | ⭘ | ⭘ | ⭘ | ⭘ | ⭘ | ⭘ | ⭘ |
| g | They explain potential side-effects before giving me a treatment | ⭘ | ⭘ | ⭘ | ⭘ | ⭘ | ⭘ | ⭘ |
| h | I am confident that I am receiving appropriate care for my cough now | ⭘ | ⭘ | ⭘ | ⭘ | ⭘ | ⭘ | ⭘ |
| i | I am confident that I can tell whether I need to see them or whether I can take care of my cough myself | ⭘ | ⭘ | ⭘ | ⭘ | ⭘ | ⭘ | ⭘ |

C12a. What types of tests have you completed as part of evaluating your chronic cough? ***Please select all that apply.***

C12b. For each type of test selected, has this been **repeated** as part of evaluating your chronic cough? ***Please select yes or no for all tests selected***

|  |  | **Tests that have been completed** as part of evaluating your cough | **Has this test been repeated** as part of evaluating your cough? | |
| --- | --- | --- | --- | --- |
|  |  |  | Yes | No |
| 1 | Breathing tests (Lung function/ Spirometry) | 🞎 | ⭘ | ⭘ |
| 2 | Chest imaging (CT scan/ X-ray) | 🞎 | ⭘ | ⭘ |
| 3 | Allergy test | 🞎 | ⭘ | ⭘ |
| 4 | Sinus imaging (CT scan/ X-ray) | 🞎 | ⭘ | ⭘ |
| 5 | GI testing (Endoscopy/Barium swallow, Ph testing) | 🞎 | ⭘ | ⭘ |
| 6 | Bronchoscopy | 🞎 | ⭘ | ⭘ |
| 7 | Don’t know | ⭘ |  |  |
| 8 | None of the above | ⭘ |  |  |

C13. In an average month, how long do you spend **travelling to and from appointments** in relation to your chronic cough?

***Please write your best estimate.***

| 1 | ⭘ | _______________ hours |
| --- | --- | --- |

C14. How do you feel about how long it takes you to travel to and from appointments in relation to your chronic cough?

***Please select one answer.***

| 1 | ⭘ | Takes too long, and I struggle to be able to attend every appointment |
| --- | --- | --- |
| 2 | ⭘ | Takes too long, but I’m still able to attend every appointment quite easily |
| 3 | ⭘ | Does not take too long, but I struggle to be able to attend every appointment |
| 4 | ⭘ | Does not take too long, and I’m able to attend every appointment quite easily |

**SECTION D: CHRONIC COUGH MANAGEMENT & TREATMENT APPROACHES [SECTION HEADER – DO NOT SHOW]**

D1a. Please select all treatments you have **ever**, to the best of your knowledge, been **prescribed/recommended by a healthcare professional** to help manage **your chronic cough**

**Please select all that apply – If the name of the drug you take is in the list then please select that row. Please refer to your medication packaging if you have them to hand.**

D1b. Next, please select all treatments you are currently prescribed/recommended by a healthcare professional to help manage your chronic cough

Please select all that apply – If the name of the drug you take is in the list then please select that row. Please refer to your medication packaging if you have them to hand.

|  |  | Q1a | Q1b |
| --- | --- | --- | --- |
|  |  | EVER prescribed to manage your cough | CURRENTLY prescribed to manage cough |
| 1 | Cough suppressant (e.g. Tylenol / Panadol (paracetamol / acetaminophen); Mucinex / Robitussin (guaifenesin) | 🞎 | 🞎 |
| 2 | Neuromodulators (e.g. Neurontin (gabapentin), Lyrica (pregabalin), Elavil (amitriptyline)) | 🞎 | 🞎 |
| 3 | Proton pump inhibitors (e.g. Nexium (esomeprazole), Losec (omeprazole), Prevacid (Lansoprazole), etc.), | 🞎 | 🞎 |
| 4 | H2 blockers (e.g. Zantac (ranitidine), famotidine) | 🞎 | 🞎 |
| 5 | Nasal steroids (e.g. fluticasone propionate, Nasonex (mometasone)) | 🞎 | 🞎 |
| 6 | Inhaled steroids (e.g. Pulmicort (budesonide), Qvar (beclomethasone), Flovent (fluticasone)) | 🞎 | 🞎 |
| 7 | Oral steroids (e.g. prednisone) | 🞎 | 🞎 |
| 8 | Beta-agonists (e.g. Ventolin (salbutamol), formoterol, vilanterol) | 🞎 | 🞎 |
| 9 | Anti-histamines / Allergy relief medications (e.g. Allegra, Reactine, Claritin, etc.) | 🞎 | 🞎 |
| 10 | Morphine | 🞎 | 🞎 |
| 11 | Codeine | 🞎 | 🞎 |
| 12 | Cough drops / syrup | 🞎 | 🞎 |
| 13 | Decongestant | 🞎 | 🞎 |
| 14 | Antibiotics | 🞎 | 🞎 |
| 15 | Antidepressants | 🞎 | 🞎 |
| 16 | Sleeping aid medication | 🞎 | 🞎 |
| 17 | Speech and language therapy | 🞎 | 🞎 |
| 18 | I can’t remember the name of the treatment(s) | ⭘ | |
| 19 | Not currently prescribed any treatment(s) |  | ⭘  **[SKIP TO Q10]** |

D2. For how long have you been receiving your currently prescribed/recommended treatment for chronic cough?

***Please write your best estimate and select the relevant unit below.***

**[PN: ASK ALL CURRENTLY TAKING PRESCRIBED TREATMENT (ALL SELECTED AT D1b)]**

| _________ | ⭘ Weeks | ⭘ Months | ⭘ Years | ⭘ Don’t know |
| --- | --- | --- | --- | --- |

D3. Please select the response below which best describes your current level of satisfaction with your currently prescribed/recommended chronic cough treatment.

***Please select one answer.***

**[PN: ASK ALL CURRENTLY TAKING PRESCRIBED TREATMENT (ALL SELECTED AT D1b)]**

| 1 | ⭘ | Satisfied, AND I believe this is the best that can be achieved for my cough **[SKIP TO D5]** |
| --- | --- | --- |
| 2 | ⭘ | Satisfied, BUT I believe better control could be achieved for my cough **[SKIP TO D5]** |
| 3 | ⭘ | Not satisfied, BUT I believe this is the best that can be achieved for my cough |
| 4 | ⭘ | Not satisfied AND I believe better control could be achieved for my cough |

**PN: ASK IF CODE 3 OR 4 AT D3**

D4. Have you discussed your dissatisfaction regarding your currently prescribed/recommended chronic cough treatment with your main healthcare professional?

***Please select one answer.***

| 1 | ⭘ | Yes |
| --- | --- | --- |
| 2 | ⭘ | No |

D5. How much do you agree with the following statements in relation to your current prescribed/recommended treatment for your chronic cough?

Please rate each statement below, where 1 (strongly disagree); to 7 (strongly agree) or D/K if you don’t know or unsure

**[PN: ASK ALL CURRENTLY TAKING PRESCRIBED TREATMENT (ALL SELECTED AT D1b)]**

|  |  | 1  Strongly disagree | 2 | 3 | 4 | 5 | 6 | 7  Strongly agree | D/K |
| --- | --- | --- | --- | --- | --- | --- | --- | --- | --- |
| a | Overall, I am very satisfied with my treatment’s control of my cough | ⭘ | ⭘ | ⭘ | ⭘ | ⭘ | ⭘ | ⭘ | ⭘ |
| b | My treatment is effective at treating my cough symptoms | ⭘ | ⭘ | ⭘ | ⭘ | ⭘ | ⭘ | ⭘ | ⭘ |
| c | I know what each of my prescribed cough medications do | ⭘ | ⭘ | ⭘ | ⭘ | ⭘ | ⭘ | ⭘ | ⭘ |
| d | I am worried about short-term side effects of my cough treatment | ⭘ | ⭘ | ⭘ | ⭘ | ⭘ | ⭘ | ⭘ | ⭘ |
| e | I worry about the long-term side effects of my cough treatment | ⭘ | ⭘ | ⭘ | ⭘ | ⭘ | ⭘ | ⭘ | ⭘ |
| f | I would recommend my cough treatment to another cough patient | ⭘ | ⭘ | ⭘ | ⭘ | ⭘ | ⭘ | ⭘ | ⭘ |
| g | I do not want to take my cough treatment for the foreseeable future | ⭘ | ⭘ | ⭘ | ⭘ | ⭘ | ⭘ | ⭘ | ⭘ |
| h | I am worried about the perception of taking strong pain medication to manage my cough | ⭘ | ⭘ | ⭘ | ⭘ | ⭘ | ⭘ | ⭘ | ⭘ |
| i | I wish there was an alternative treatment to my current cough medication | ⭘ | ⭘ | ⭘ | ⭘ | ⭘ | ⭘ | ⭘ | ⭘ |

D6. Do you ever reduce or stop taking one of your prescribed/recommended cough medications without informing your main healthcare professional?

***Please select one answer.***

**[PN: ASK ALL CURRENTLY TAKING PRESCRIBED TREATMENT (ALL SELECTED AT D1b)]**

| 1 | ⭘ | All of the time |
| --- | --- | --- |
| 2 | ⭘ | Often |
| 3 | ⭘ | Sometimes |
| 4 | ⭘ | Rarely **[SKIP TO D8]** |
| 5 | ⭘ | Never **[SKIP TO D8]** |

**PN: ASK IF CODE 1,2 OR 3 AT D6**

D7. What are the reasons for reducing / stopping one of your currently prescribed/recommended cough medications?

***Please select all that apply.***

| 1 | 🞎 | I forget to take my medication |
| --- | --- | --- |
| 2 | 🞎 | I don’t feel like it is working |
| 3 | 🞎 | I don’t think I need to take the medication as frequently as prescribed |
| 4 | 🞎 | I control my symptoms with non-prescription / over-the-counter treatments |
| 5 | 🞎 | I control my symptoms with non-drug treatments |
| 6 | 🞎 | I don’t like the side effects caused by taking the medication |
| 7 | 🞎 | I prefer not to take medication |
| 8 | 🞎 | I don’t feel like I have enough information about the medication |
| 9 | 🞎 | A friend taking this medication experienced negative effects |
| 10 | 🞎 | I heard about its side effects on Facebook/other social media |
| 11 | 🞎 | My medication instructions are unclear |
| 12 | 🞎 | Other reason (please specify _______________________) |

D8. Do you or your family pay anything towards the cost of your **prescribed/recommended** cough medications?

***Please select one answer.***

**[PN: ASK ALL CURRENTLY TAKING PRESCRIBED TREATMENT (ALL SELECTED AT D1b)]**

| 1 | ⭘ | Yes |
| --- | --- | --- |
| 2 | ⭘ | No **[SKIP TO Q10]** |

**PN: ASK IF CODE 1 AT D8**

D9. How much did you, or your family, have to pay for your **prescribed/recommended medication(s)** for chronic cough in the **past month**?

***Please write your best estimate.***

| 1 | ⭘ | $ ______________ |
| --- | --- | --- |
| 2 | ⭘ | Don’t know |

D10. Have you ever not taken a prescription/recommended medication for your cough because of cost?

***Please select one answer.***

| 1 | ⭘ | Yes |
| --- | --- | --- |
| 2 | ⭘ | No |
| 3 | ⭘ | Not applicable |

D11. Other than a cure for your chronic cough, what are the **THREE most important benefits** a cough treatment could provide? *Please assign rankings (1 to 3) with 1 being the most important, 2 the next most important etc.*

**PN: PROGRAM A DRAG AND DROP EXERCISE**

| 1 |  | Preventing worsening of my chronic cough |
| --- | --- | --- |
| 2 |  | Improving my cough symptoms more quickly |
| 3 |  | Improving my mood (less anxiety, depressed feelings) |
| 4 |  | Reducing background pain levels |
| 5 |  | Reducing general tiredness and fatigue |
| 6 |  | Reducing urinary stress incontinence |
| 7 |  | Reducing impact on my spouse / family members |
| 8 |  | Improving my sleep quality |
| 9 |  | Increasing ability to attend work |
| 10 |  | Increasing productivity when at work |
| 11 |  | Improving my ability to socialise with friends |
| 12 |  | Improving my ability to travel |
| 13 |  | Reducing number of hospital stays / days spent in hospital |
| 14 |  | Increase the number of days free from coughing |
| 15 |  | Other (please specify below) |
|  |  | ____________________________________________ |

D12. Overall, how would you describe your involvement in treatment decisions for your chronic cough? ***Please select one answer.***

| 1 | ⭘ | My physician makes all treatment decisions without my involvement |
| --- | --- | --- |
| 2 | ⭘ | My physician discusses treatment options with me, but it is ultimately their decision |
| 3 | ⭘ | My physician discusses treatment options with me, and we make the decision together |
| 4 | ⭘ | My physician discusses treatment options with me, and I make the final decision |

D13. Have you ever chosen to stop seeking further medical attention for your cough at any point, because of a lack of success in treating it?

***Please select one answer.***

| 1 | ⭘ | Yes |
| --- | --- | --- |
| 2 | ⭘ | No |

D14. Have you ever purchased any **non-prescription / over-the-counter medications** (e.g. cough drops/syrups, anti-histamines, nasal decongestants etc.) at the pharmacy to treat your cough?

***Please select one answer.***

| 1 | ⭘ | Yes |
| --- | --- | --- |
| 2 | ⭘ | No **[SKIP TO D16]** |

**PN: ASK IF CODE 1 AT D14**

D15. If yes, how much have you spent on **non-prescription / over-the-counter medications** at the pharmacy to treat your cough in the **past month**?

***Please write your best estimate.***

| 1 | ⭘ | $ ______________ |
| --- | --- | --- |
| 2 | ⭘ | Don’t know |

D16. In an average month, how much do you pay out of your own money for the other expenses below, in relation to your chronic cough (excluding any money you may receive to help you with these costs from your healthcare coverage)?

*Please write your best estimate (in $ per month).*

|  | **Type of expense** | **Approximate cost (*$/*monthly)** |
| --- | --- | --- |
| a | Travel to doctor / hospital / medical appointments | $______________ |
| b | Parking fees (i.e. at the pharmacy, or hospital / clinic) | $______________ |
| c | Tests or labs, i.e. blood tests, scans, allergy test, etc. | $______________ |
| d | Other costs incurred (excluding costs for any medications or therapy related to your cough) | $______________ |

D17. Have you ever tried **home remedies** to treat your chronic cough, such as herbal supplements, neti-pots, saline sprays, Chinese medicine etc.?

***Please select one answer.***

| 1 | ⭘ | Yes |
| --- | --- | --- |
| 2 | ⭘ | No |

D18. Do you receive any professional support (e.g. psychiatrist, psychologist, counselling support) to help you manage any emotional aspects of your chronic cough?

***Please select one answer.***

| 1 | ⭘ | Yes **[SKIP TO D20]** |
| --- | --- | --- |
| 2 | ⭘ | No |

D19. Do you sometimes feel that coughing has resulted in people avoiding you or distancing themselves, thereby negatively impacting your ability to socialize?

***Please select one answer.***

| a | Never | ⭘ |
| --- | --- | --- |
| b | Rarely | ⭘ |
| c | Occasionally | ⭘ |
| d | Often | ⭘ |

D20. Do you find that Chronic Cough affects your emotional or mental well-being?

***Please select one answer.***

| a | Not at all | ⭘ |
| --- | --- | --- |
| b | Somewhat | ⭘ |
| c | Significantly | ⭘ |
| d | Substantially | ⭘ |

**PN: ASK IF CODE 2 AT D18**

D21. Do you wish you were able to receive any / additional professional support (e.g. psychiatrist, counselling support) for any emotional aspects of your chronic cough?

***Please select one answer.***

| 1 | ⭘ | Yes |
| --- | --- | --- |
| 2 | ⭘ | No |

D22. How comfortable do you feel discussing any **emotional** aspects of your chronic cough on your personal / private life with your doctor?

Please rate the statement below, where 1 = very uncomfortable and 7 = very comfortable.

| 1  Very uncomfortable | 2 | 3 | 4 | 5 | 6 | 7  Very comfortable |
| --- | --- | --- | --- | --- | --- | --- |
| ⭘ | ⭘ | ⭘ | ⭘ | ⭘ | ⭘ | ⭘ |

**SECTION E: CHRONIC COUGH MANAGEMENT & TREATMENT APPROACHES [SECTION HEADER – DO NOT SHOW]**

E1. As a result of your chronic cough, have you ever…

***Please select one answer per row***

|  |  | **Yes** | **No** | **N/A** |
| --- | --- | --- | --- | --- |
| a | Changed from full-time to part-time employment? | ⭘ | ⭘ | ⭘ |
| b | Reduced your hours at work? | ⭘ | ⭘ | ⭘ |
| c | Seriously considered reducing your hours at work (even if you did not)? | ⭘ | ⭘ | ⭘ |
| d | Taken early retirement? | ⭘ | ⭘ | ⭘ |
| e | Voluntarily stopped working in your job? | ⭘ | ⭘ | ⭘ |
| f | Been fired/laid off or made to stop working in your job? | ⭘ | ⭘ | ⭘ |
| g | Considered terminating your job (even if you did not)? | ⭘ | ⭘ | ⭘ |
| H | Declined a promotion? | ⭘ | ⭘ | ⭘ |

E2. To what extent do you feel chronic cough has impacted on the following aspects of your career?

Please rate the following options, where 1 (not at all); to 7 (a great deal)

|  |  | 1  Not at all | 2 | 3 | 4 | 5 | 6 | 7  A great deal | Not applicable |
| --- | --- | --- | --- | --- | --- | --- | --- | --- | --- |
| a | Chronic cough has prevented me from progressing further in my career | ⭘ | ⭘ | ⭘ | ⭘ | ⭘ | ⭘ | ⭘ | ⭘ |
| b | Chronic cough has impaired my ability to keep a job | ⭘ | ⭘ | ⭘ | ⭘ | ⭘ | ⭘ | ⭘ | ⭘ |
| c | Chronic cough has prevented me from finding a suitable job | ⭘ | ⭘ | ⭘ | ⭘ | ⭘ | ⭘ | ⭘ | ⭘ |
| d | Chronic cough has limited me to certain careers (e.g. cannot do heavy manual work or jobs that involve extreme changes of temperature, concentration or long hours of not moving such as office work) | ⭘ | ⭘ | ⭘ | ⭘ | ⭘ | ⭘ | ⭘ | ⭘ |
| e | I think chronic cough has caused me to be turned down at job interviews | ⭘ | ⭘ | ⭘ | ⭘ | ⭘ | ⭘ | ⭘ | ⭘ |

[WPAI - **DO NOT SHOW**]

E3. The following questions ask about the effect of your chronic cough on your ability to work and perform normal daily activities. ***Please fill in the blanks or select an answer, as indicated.***

1. Are you currently in paid employment?

| 1 | ⭘ | Yes |
| --- | --- | --- |
| 2 | ⭘ | No **[IF NO, SKIP TO E8]** |

The next questions refer to the past seven days, not including today.

**PN: ASK IF CODE 1 AT E3**

E4. During the past seven days, how many hours did you miss from work because of problems associated with your chronic cough?

*Include hours you missed on sick days, times you went in late, left early, etc., because of your chronic cough. Do not include time you missed to participate in this study.*

| 1 | ⭘ | _______________ hours |
| --- | --- | --- |

**PN: ASK IF CODE 1 AT E3**

E5. During the past seven days, how many hours did you miss from work because of any other reason, such as annual leave, holidays, time off to participate in this study?

| 1 | ⭘ | _______________ hours |
| --- | --- | --- |

**PN: ASK IF CODE 1 AT E3**

E6. During the past seven days, how many hours did you actually work?

| 1 | ⭘ | _______________ hours **[IF ‘0’, SKIP TO E8]** |
| --- | --- | --- |

**PN: ASK IF CODE 1 AT E3 AND >0 AT E6**

E7. During the past seven days, how much did your chronic cough affect your productivity while you were working?

*Think about days you were limited in the amount or kind of work you could do, days you accomplished less than you would like, or days you could not do your work as carefully as usual. If your chronic cough affected your work only a little, choose a low number. Choose a high number if your chronic cough affected your work a great deal.*

| *Consider only how much your chronic cough  affected productivity while you were working* | | | | | | | | | | | | |
| --- | --- | --- | --- | --- | --- | --- | --- | --- | --- | --- | --- | --- |
| *My chronic cough had no effect on my work* | *__________________________________________________* | | | | | | | | | | | *My chronic cough completely prevented me  from working* |
|  | *0* | *1* | *2* | *3* | *4* | *5* | *6* | *7* | *8* | *9* | *10* |  |
|  |  | | | | | | | | | | |  |

**PN: ASK ALL**

E8. During the past seven days, how much did your chronic cough affect your ability to perform your normal daily activities, excluding your job?

*By normal activities, we mean the usual activities you perform, such as working around the house, shopping, childcare, exercising, studying, etc. Think about times you were limited in the amount or kind of activities you could perform and times you accomplished less than you would like. If your chronic cough affected your activities only a little, choose a low number. Choose a high number if your chronic cough affected your activities a great deal.*

| *Consider only how much your chronic cough affected your ability*  *to perform your normal daily activities, excluding your job* | | | | | | | | | | | | |
| --- | --- | --- | --- | --- | --- | --- | --- | --- | --- | --- | --- | --- |
| *My chronic cough had no effect on my daily activities* | *__________________________________________________* | | | | | | | | | | | *My chronic cough completely prevented me  from performing my daily activities* |
|  | *0* | *1* | *2* | *3* | *4* | *5* | *6* | *7* | *8* | *9* | *10* |  |

**SECTION F: DEMOGRAPHICS**

F1. How long have you lived in Canada?

| Since I was born | 1 |
| --- | --- |
| More than 20 years | 2 |
| Between 15 & 20 years | 3 |
| Between 10 & 14 years | 4 |
| Between 5 & 9 years | 5 |
| Less than 5 years | 6 |
| I prefer not to answer | 9 |

F2. What is your ethnicity? *Please select all that apply.*

| South Asian (e.g. East Indian, Pakistani, Sri Lankan, etc) | 1 |
| --- | --- |
| Southeast Asian (e.g. Vietnamese, Cambodian, Malaysian, etc.) | 2 |
| West Asian (e.g. Iranian, Afghan etc.) | 3 |
| White/Caucasian | 4 |
| Indigenous | 5 |
| Black | 6 |
| Latin American | 7 |
| Chinese | 8 |
| Korean | 9 |
| Japanese | 10 |
| Other (specify) ___________ | 11 |
| I prefer not to answer | 99 |

F3. Which of the following best describes where you currently live?

| Large urban centre with more than 1 million inhabitants | 1 |
| --- | --- |
| Large urban centre with >500,000 but < 1 million inhabitants | 2 |
| Medium urban centre (100,000 to 500,000 inhabitants) | 3 |
| Small urban centre (10,000 - 99,000 inhabitants) | 4 |
| Rural/ small suburb (<10,000 inhabitants) | 5 |

F4. What is your current marital status?

| Single / never married | 1 |
| --- | --- |
| Married | 2 |
| Common law | 3 |
| Separated | 4 |
| Divorced | 5 |
| I prefer not to answer | 99 |

F5. Including yourself, how many people live in your household, counting adults and children?

_____ # people

O One person (myself)

**ASK IF >1 AT F5**

F6. How many children in each of the following age groups (if any) do you have living with you in your household?

| 5 years of age or under | ___# |
| --- | --- |
| 6 to 12 years of age | ___# |
| 13 to 17 years of age | ___# |
| 18 years of age or older | ___# |
| I do not have any children living in my household | 98 |
| I prefer not to answer | 99 |

F7. What is the last year of education that you have completed?

| Elementary (7 years or less) | 1 |
| --- | --- |
| High school, general or vocational (8 to 12 years) | 2 |
| College (pre-university, technical training, certificate, accreditation) | 3 |
| University certificates and diplomas | 4 |
| University Bachelor (including classical studies) | 5 |
| University Master's degree | 6 |
| University Doctorate (PhD) | 7 |
| I prefer not to answer | 99 |

F8. What is your current employment status?

| Working full-time | 1 |
| --- | --- |
| Working part-time | 2 |
| Homemaker, no outside employment | 3 |
| Student | 4 |
| Unemployed | 5 |
| Retired | 6 |
| Disabled | 7 |
| Other | 98 |
| I prefer not to answer | 99 |

F9. Which of the following best describes your current health insurance coverage?

| Public / provincial coverage | 1 |
| --- | --- |
| Private insurance | 2 |
| No coverage | 3 |
| Don’t know | 98 |
| I prefer not to answer | 99 |

F10. Which of the following categories best reflects your total annual household income?

| Less than $20,000 | 1 |
| --- | --- |
| $20,000 to $39,999 | 2 |
| $40,000 to $59,999 | 3 |
| $60,000 to $79,999 | 4 |
| $80,000 to $99,999 | 5 |
| $100,000 to $119,999 | 6 |
| $120,000 or more | 7 |
| I prefer not to answer | 99 |

F11. Please specify your height.

*Please mention either in centimeters* *or feet and inches.*

| 1 | ⭘ | ______ centimeters (cm) |
| --- | --- | --- |
| 2 | ⭘ | ______ feet _____ inches |
| 99 | ⭘ | I prefer not to answer |

F12. Please specify your weight.

*Please mention either in kilograms or pounds.*

| 1 | ⭘ | ______ kilograms (kg) |
| --- | --- | --- |
| 2 | ⭘ | ______ Pounds (lbs.) |
| 99 | ⭘ | I prefer not to answer |

**Those are all the questions we have. Thank you for participating in this study.**
